# Synthesis and Biological Evaluation of 2,5-Bis(alkylamino)-1,4-benzoquinones

**DOI:** 10.3390/molecules15085629

**Published:** 2010-08-13

**Authors:** Luiz Cláudio Almeida Barbosa, Ulisses Alves Pereira, Célia Regina Alvares Maltha, Róbson Ricardo Teixeira, Vânia Maria Moreira Valente, José Roberto Oliveira Ferreira, Letícia Veras Costa-Lotufo, Manoel Odorico Moraes, Cláudia Pessoa

**Affiliations:** 1 Department of Chemistry, Federal University of Viçosa. Av. P.H. Rolfs, CEP 36570-000, Viçosa, MG, Brasil; 2 Department of Physiology and Pharmacology, Federal University of Ceará, St. Coronel Nunes de Melo, 1127, CEP 60431-970, Fortaleza, CE, Brasil

**Keywords:** quinones, herbicide, cytotoxicity

## Abstract

A series of twelve 2,5-bis(alkylamino)-1,4-benzoquinones were prepared in yields ranging from 9–58% *via* the reaction between *p*-benzoquinone and various amines. The structures of the synthesized compounds were confirmed by IR, ^1^H- and ^13^C-NMR and MS analyses. The phytotoxicity of the 2,5-bis(alkylamino)-1,4-benzoquinones was evaluated against two crop species, *Cucumis sativus* and *Sorgum bicolor*, at 1.0 × 10^-3^ mol/L. In general, the quinones displayed inhibitory effects on the dicotyledonous species *C. sativus* (7–74%). On the other hand stimulatory effects were observed on *S. bicolor* (monocotyledonous). Similar results were observed in the biological assays carried out with the weed species *Ipomoea grandifolia* (dicotyledonous) and *Brachiaria decumbens* (monocotyledonous). In addition, the cytotoxicity of the 2,5-bis(alkylamino)-1,4-benzoquinones was assayed against HL-60 (leukemia), MDA-MB-435 (melanoma), SF-295 (brain) and HCT-8 (colon) human cancer cell lines and human peripheral blood mononuclear cells (PBMC), as representatives of healthy cells, using a MTT and an Alamar Blue assay. Compound **12** was the most active, displaying cytotoxicity against all cancer cell lines tested.

## 1. Introduction

Plant roots perform a variety of functions such as mechanical support, water/nitrogen uptake, and production of exudates. It is known that roots are capable of producing and secreting compounds (exudates) into the rhizosphere. Root exudation includes release of ions, free oxygen and water, enzymes, mucilage, and a range of organic compounds (primary and secondary metabolites) [1,2,3,4].

**Figure 1:**
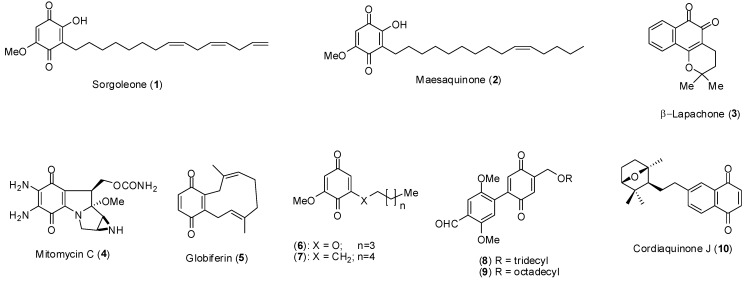
Structures of several benzoquinones.

Various organic compounds present in root exudates are involved in chemical-mediated plant-plant or allelopathy interactions. Allelopathy is concerned with the chemical interactions that occur among plants and is mediated by the release of allelochemicals into the rhizosphere [5,6]. The study of such interactions has led to the discovery of several phytotoxic substances that have potential use as herbicides [7], or could be used as lead structures for the development of more active compounds [8]. Among such substances is a compound called sorgoleone (**1**, Figure 1), isolated from root exudates of *Sorghum bicolor* [9] and characterized as 2-hydroxy-5-methoxy-3-[(8*Z*,11*Z*)-pentadeca-8,11,14-trienyl]- cyclohexa-2,5-dien-1,4-dione. 

Since its discovery, it has been demonstrated that **1** is a potent inhibitor of chlorophyll formation in *Lemma minor* L., and it also inhibits the growth of several grass and broadleaf weeds at concentrations as low as 10 μmol/L [10]. Further investigations revealed that **1** is an effective inhibitor of electron transfer between Q_A_^-^ to Q_B_ at the reducing side of photosystem II [11] and also of the mitochondrial electron transport [12,13]. During *in vitro* assays, **1** has been shown to be more active than the commercial herbicide atrazine in inhibiting photosystem II [14]. This quinone also causes disturbance of plasma H^+^-ATPase activity in root cells [15]. 

It has also been demonstrated that several benzoquinones displayed cytotoxic effects. For example, the naturally occurring maesaquinone (**2**, Figure 1) exhibited *in vitro* cytotoxicity against several solid tumor cells [16]. β-Lapachone (**3**, **Figure 1**), has a diversity of useful biological activities against various cancer cell lines such as human ovarian and prostate tumors and, at lower doses, is a radiosensitizer of several human cancer cell lines [17]. The quinone mitomycin C (**4**, Figure 1) has been used in chemotherapy against certain solid tumors [18,19]. More recently, the biological profile of globiferin (**5**, Figure 1) a terpenoid benzoquinone isolated from root extracts of *Cordia globifera* was investigated [20]. It was found that this compound displays cytotoxic activity against NCI-H187 cell line. The biological profile of cordiaquinone J (**10**, Figure 1), a 1,4-naphthoquinone isolated from the roots of *Cordia leucocephala*, has been shown to present antiproliferative effects related to reactive oxygen species (ROS) generation [21].

In the last few years, we have targeted various natural products as lead structures towards the development of plant growth regulators [22,23,24,25,26,27,28,29], including sorgolene (**1**) [30,31]. In this context, several sorgoleone derivatives, such as compounds **6**-**9** (Figure 1), were prepared and subsequently evaluated against crop and weed species. It has been found that the synthetic analogues **6** and **7** are more effective than **1**. Moreover, while compound **6** inhibited the root development of the weed species *Euphorbia heterophylla* and *Brachiaria decumbens*, compound **7** was effective in inhibiting the development of the aerial parts and roots of *B. decumbens*, an aggressive weed commonly found in several crop plantations in Brazil. When tested against *E. heterophylla*, the aryl-*p*-benzoquinone **8** caused 34.2 and 76.5% inhibition of the aerial part and roots, respectively. The quinone **9** significantly inhibited the aerial parts (5l.7%) and roots (85.2%) of the weed species *Ipomoea grandifolia*.

In continuation to our studies in this area we report in this paper the preparation and investigation of the phytotoxic effects of twelve 2,5-bis(alkylamino)-1,4-benzoquinone analogues of sorgoleone (**1**). Considering the cytotoxic effects reported in the literature for several benzoquinones, we also evaluated the cytotoxicity of these analogues against several human cancer cell lines and peripheral blood mononuclear cells (PMBC). The results of the cytotoxicity screening are also discussed.

## 2. Results and Discussion

### 2.1. Synthesis of 2,5-diamino-p-benzoquinones

**Scheme 1:**
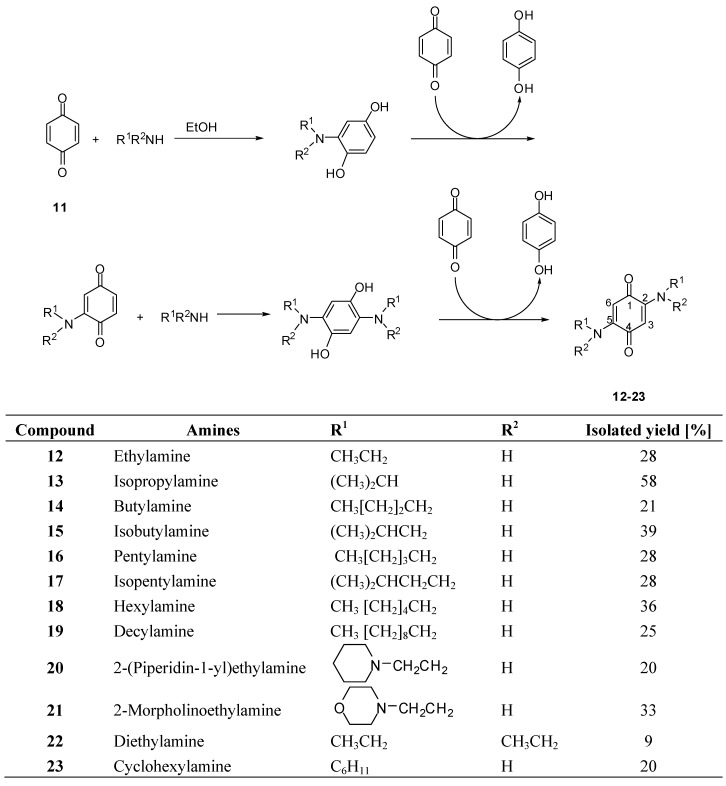
Synthesis of 2,5-bis(alkylamino)-1,4-benzoquinones **12**-**23**.

Compounds **12**-**23** were synthesized by the reaction between *p*-benzoquinone **11** and various amines [32,33,34]. A 3:2 molar ratio of benzoquinone/amine was required due to successive reductions and oxidations involved in the formation of the products as shown in Scheme 1. The reaction was carried out first in dry EtOH and later it was found that slightly better yields were obtained when wet EtOH containing 2% (*v/v*) of H_2_O was used. Although no detailed investigation on the mechanism was carried out, we believe that H_2_O might enhance the reactivity and polarizability of the quinone carbonyl group facilitating the nucleophilic addition step [33].

After column chromatography purification, the 2,5-bis(alkylamino)-1,4-benzoquinones were obtained as red compounds. Bayen and co-workers [32] reported the preparation of several 2,5-bis(alkylamino)-1,4-benzoquinones in good yields employing similar conditions used in the present work. In our hands, however, benzoquinones **12**-**23** were obtained in only low to fair yields (Scheme 1). This fact can possibly be attributed to polymerization side reactions as previously reported [35]. These results are also consistent with a great quantity of base line material observed during the column chromatographic purification. 

The IR spectra of compounds **12-23** exhibited strong absorptions in the 1,613 to 1,550 cm^-1^ range due to carbonyl stretchings. The vinylic H-atoms in these quinones were characterized in the NMR spectrum by signals observed at 5.3–5.4 ppm, while resonance signals for the C=O groups were observed at 176-180 ppm. The expected molecular formulae were confirmed by high resolution ESI-MS.

### 2.2. Phytotoxic activity

In a preliminary screen carried out on Petri dishes the effect of 2,5-bis(alkylamino)-1,4-benzoquinones **12-23** on the radicle growth of *S. bicolor* and *C. sativus* was evaluated at a concentration of 1.0 × 10^-3^ mol/L (Table 1).

**Table 1:** Effect of 2,5-bis(alkylamino)-1,4-benzoquinones on the radicle growth of *C. sativus *and *S. bicolor* at 1.0 × 10^-3^ mol/L.

Compound	*Cucumis sativus*				*Sorghum bicolor*			
	24 h		48 h		24 h		48 h	
	Radiclelength [cm]^a^	Inhibition [%]	Radiclelength [cm]^a^	Inhibition [%]	Radiclelenght [cm]^a^	Inhibition [%]	Radiclelength [cm]^a^	Inhibition [%]
**12**	1.40 def	42	1.94 fg	59	0.87 cd	36	1.04 fg	30
**13**	1.75 cde	28	3.91 bcd	18	1.90 ab	-40	3.50 abc	-135
**14**	2.17 abc	10	4.87 a	-2	1.69 ab	-24	3.70 ab	-148
**15**	2.02 abc	17	3.43 de	28	1.14 bcd	17	1.87 defg	-26
**16**	2.02 abc	17	4.24 abcd	11	1.52 abc	-12	1.91 defg	-28
**17**	1.89 bcd	22	2.73 ef	43	1.17 bcd	14	2.25 bcdef	-51
**18**	1.25 ef	48	1.25 g	74	0.49 d	64	0.51 g	66
**19**	2.26 abc	7	3.73 ef	22	1.81 ab	-33	3.81 a	-156
**20**	1.85 cd	24	2.33 f	51	1.23 bcd	10	2.07 cdef	-39
**21**	2.46 a	-2	4.41 abc	7	2.09 a	-54	3.21 abcd	-115
**22**	1.16 f	52	1.79 fg	62	0.85 cd	38	0.92 fg	38
**23**	2.10 abc	13	4.41 abc	7	1.47 abc	-8	2.82 abcde	-89
**Control**	2.42 ab		4.76 ab		1.36 abc		1.49 efg	

^a^ Means, in the same column, with the same letter are not significantly different at *P* = 0.05% by Tukey’s test.

This type of biological assay is commonly used as a general screening for identifying potential phytotoxic substances [36]. With the exception of compound **14**, all of the remaining substances inhibited the radicle growth of the dicotyledonous species *C. sativus* after 48 h. Compound **18 **displayed the highest effectiveness against this species, causing 74% inhibition. A simple correlation could not be found between the length of the side chain and the observed biological activity of compounds **12**, **13**, **14**, **16**, **18**, and **19**. Comparing quinones **14** (-2%)and **15** (28%) as well as **16** (11%) and **17** (43%) it can be observed that the compounds with an unbranched side chain exhibited major inhibitory activity in relation to the branched side chain. Considering compounds **20**, **21**, and **23**, which present cyclic groups in their side chain portions, the highest inhibitory effect was associated with compound **20**. From the results presented in Table 1 it is clear that while inhibitory effects were observed for *C. sativus*, after 48 hours radicle promotion was noticed with the compounds for the monocotyledonous species *S. bicolor*. Exceptions to this generalization were the substances **12**, **18**, and **22**.

The effect of the compounds **12-23 **on the radicle growth of the weed species *Ipomoea grandifolia* (dicotiledonous) and *Brachiaria decumbens* (monocotiledonous) was subsequently investigated. As presented in Table 2, differential effects were observed between the two weed species. As a general trend, inhibitory effects were observed for *I. grandifolia* while stimulation was noticed for *B. decumbens*. 

**Table 2:** Effect of 2,5-bis(alkylamino)-1,4-benzoquinones on the radicle growth of *I. grandifolia* and *B. decumbens* at 1.0 × 10^-3^ mol/L.

Compound	*Ipomoea grandifolia*				*Brachiaria decumbens*			
	24 h		48 h		24 h		48 h	
	Radiclelength [cm]^a^	Inhibition [%]	Radiclelength [cm]^a^	Inhibition [%]	Radiclelenght [cm]^a^	Inhibition [%]	Radiclelength [cm]^a^	Inhibition [%]
**12**	0.91 de	48	0.91 d	67	0.87 cd	47	1.06 bcd	43
**13**	1.19 cd	32	2.47 ab	10	1.82 a	-11	2.90 a	-56
**14**	1.41 abc	20	2.74 a	0	1.69 ab	-3	3.07 a	-65
**15**	1.43 abc	19	2.39 ab	13	1.48 abc	10	2.35 ab	-26
**16**	1.48 abc	16	2.41 ab	12	1.10 bcd	33	1.50 bcd	19
**17**	1.23 cd	30	1.49 bcd	46	1.59 abc	3	2.21 ab	-19
**18**	0.94 de	47	0.94 d	66	0.45 d	73	0.45 d	76
**19**	1.69 ab	4	2.25 abc	18	1.67 ab	-2	2.98 a	-60
**20**	1.22 cd	31	1.57 bcd	43	1.51 abc	8	1.93 abc	-4
**21**	1.32 bcd	26	2.40 ab	12	1.80 ab	-10	2.82 a	-51
**22**	0.77 e	56	1.35 cd	51	0.65 d	60	0.86 cd	54
**23**	1.35 bc	23	2.72 a	1	1.73 ab	-5	3.02 a	-62
**Control**	1.76 a		2.72 a		1.64 ab		1.86 abc	

^a^ Means, in the same column, with the same letter are not significantly different at *P* = 0.05% by Tukey’s test

### 2.3. ATP assay

Considering a previous report on the literature concerning the influence of 2,5-diaminobenzo-quinones on ATP synthesis [37], we assessed the ability of compounds **12**, **13**, and **23** to interfere with this process. The choice of these substances was made based on the higher availability of them in our laboratories. These three compounds displayed inhibitory activity on the ATP synthesis in isolated spinach chloroplasts. The best inhibitory activity was observed for compound **13** which presented an IC_50_ equal to 150 µM (data not shown).

### 2.4. Anti-tumor activity

The anti-tumor activity of the 2,5-bis(alkylamino)-1,4-benzoquinone sorgoleone analogues **12-14**, **16-23** was evaluated against four human cancer cell lines: HL-60 (promyelocytic leukemia), HCT-8 (colon), SF-295 (central nervous systems) and MDA-MB-435 (melanoma), obtained from the National Cancer Institute (Bethesda, MD, USA), using the MTT assay as previously described [38]. To investigate the selectivity of different compounds toward normal proliferating cells, an Alamar Blue assay was performed with PBMC after 72 h of drug exposure. The results of the IC_50_ data [μg/mL] for the antitumor activities are presented in Table 3. Doxorubicin was used as positive control. After 72 h, compounds **12** and **20** exhibited cytotoxicity against all tumor cell lines tested. Compound **12 **was a more potent proliferation inhibitor (IC_50_ values in the range 2.0–6.0 µg/mL) than compound **20**. The other compounds investigated were not able to significantly inhibit cell growth under the assay conditions, presenting IC_50_ values higher than 25 µg/mL.

The cytotoxic activity of the compounds **12** and **20** against normal cells (PBMC) was assessed by Alamar Blue assay [39]. This assay was chosen because of its low toxicity to normal cells [40]. It was found that compound **20** was cytotoxic, with an IC_50_ value 21.8 µg/mL. Compound **12** showed no activity against PBMC at the highest concentration tested (>25 µg/mL). This compound exhibited the most selective cytotoxicity against the tumor lines and is not toxic to normal cells. It is important to emphasize that selectivity is one important feature towards the development of new antitumoral drugs [41].

**Table 3:** Cytotoxic activity of 2,5-bis(alkylamino)-1,4-benzoquinone analogues to sorgoleone.

Compound	Cells^a^ IC_50_^b^ [µg/mL]; Confident interval					
	HL-60	SF-295	HCT-8	MDA-MB-435	PBMC	
**12**	2.3(1.3-3.9)	6.0 (4.6-7.9)	5.2(2.4-11.2)	5.6(3.8-8.4)	>25	
**13**	>25	>25	>25	>25	Nd	
**14**	>25	>25	>25	>25	Nd	
**16**	>25	>25	>25	>25	Nd	
**17**	>25	>25	>25	>25	Nd	
**18**	>25	>25	>25	>25	Nd	
**19**	>25	>25	>25	>25	Nd	
**20**	20.3(17.6-23.4)	11.3(8.6-14.9)	13.5(9.1-20.1)	21.5(18.7-24.8)	21,8 (15,6-30,1)	
**21**	>25	>25	>25	>25	Nd	
**22**	>25	>25	>25	>25	Nd	
**23**	>25	>25	>25	>25	Nd	
**Doxorrubicin**	0.02(0.01-0.02)	0.23(0.19-0.25)	0.01(0.01-0.02)	0.48(0.34-0.66)	0,96 (0,51-1,71)	

^a^ Cells were plated in 96-well plates incubated under a 5% CO_2_ atmosphere at 37 °C for 72 h in the presence of pure compounds (0.39-25 μg/mL). Each concentration was tested in triplicate and the analyses were performed in duplicate; ^b^ Data are presented as IC_50_ [μg/mL] values and 95% confidence interval (given in parentheses) obtained from at least three independent experiments. Compound **15** was not evaluated due to limited amount. Nd: not determined.

The quinone structural motif is present in many anticancer drugs such as anthracyclines (daunorubicin, doxorubicin), mitomycin and mitoxantrone, which are used clinically in the therapy of solid tumors [42]. The mechanisms by which quinones cause these effects can be quite complex. Quinones are Michael acceptors, and cellular damage can occur through alkylation of crucial cellular proteins and/or DNA. Alternatively, quinones are highly redox active molecules which can, by intermediacy of their semiquinone radicals, lead to formation of reactive oxygen species (ROS), which can cause severe oxidative stress within cells through the formation of oxidized cellular macromolecules, including lipids, proteins, and DNA. ROS can also activate a number of signaling pathways. Additionally, quinones, including azaquinones, can work as DNA intercalators, inhibitors of topoisomerases and of some enzymes of the mitochondrial electron transfer chain.

## 3. Experimental

### 3.1. General

Hydroquinone and amines were purchased from Aldrich (Milwaukee, WI, USA) Reagents and solvents were purified, when necessary, according to procedures described by Perrin and Armarego [43]. *p*-Benzoquinone **11** was synthesized from hydroquinone employing a previously described procedure [44]. Analytical thin layer chromatography analyses were conducted on aluminum backed pre-coated silica gel plates. Column chromatography was performed on silica gel (60–230 mesh) and eluting with hexane:diethyl ether mixtures. Melting points are uncorrected and were obtained on a MQAPF-301 apparatus (Microquimica, Brazil). IR spectra were recorded on a Perkin Elmer Paragon 1000 FTIR spectrophotometer using KBr discs (1% *w/w*) and scanning from 400 to 4,000 cm^-1^. The ^1^H- and ^13^C-NMR spectra were recorded on a Varian Mercury 300 instrument (at 300 MHz and 75 MHz, respectively), using deuterated CHCl_3_ as solvent and tetramethylsilane (TMS) as internal standard (*δ* = 0); coupling constants (*J*) in Hz. MS were recorded on a Shimadzu GCMS-QP5050A instrument under electron impact (70 eV) conditions. HRMS data were recorded under ESI conditions on a Bruker MicroToF spectrometer (resolution = 10,000 FWHM) using a lock-spray source. The lock-mass used for calibration was tetraoctylammonium bromide in positive ion mode.

### 3.2. Synthesis of compounds *12-16*, *18-23*, exemplified by the synthesis of *2*,5-bis(isopentylamino)-1,4-benzoquinone (*17*)

To a round-bottomed flask (125 mL) were added *p*-benzoquinone **11** (500 mg, 4.63 mmol) and EtOH 98% (*v/v*) (10 mL). After complete dissolution of **11**, isopentylamine (268 mg, 3.08 mmol) dissolved in EtOH 98% (*v/v*) (6 mL) was added slowly. The mixture was stirred at r.t. until complete consumption of the starting material (TLC analysis). The solvent was removed under reduced pressure and the residue was purified by silica gel column chromatography eluting with 1:3 (*v/v*) hexane/CH_2_Cl_2_ to give compound **17** as red crystals in 28% yield (122 mg, 0.44 mmol). Mp 167.3–169.7 ºC; IR: 3,264 (NH), 2,956, 2,870, 1,642, 1,549, 1,495, 1,459, 1,364, 1,283, 1,231, 1,071, and 812 cm^-1^; ^1^H-NMR δ: 6.59 (bs, NH); 5.30 [s, H-C(3), H-C(6)]; 3.16 [q, *J* = 6.9 Hz, 2 H-C(1′), 2 H-C(1′′)]; 1.61-1.72 [m, H-C(3′), H-C(3′′)]; 1.54 [q, *J* = 6.9 Hz, 2 H-C(2′), 2 H-C(2′′)]; 0.93 [d, *J* = 6.9 Hz, 3 H-C(4′), 3 H-C(5′), 3 H-C(4′′), 3 H-C(5′′)]; ^13^C-NMR δ: 176.1 [C(1), C(4)]; 149.4 [C(2), C(5)]; 90.6 [C(3), C(6)]; 38.9 [C(1′), C(1′′)]; 35.0 [C(2′), C(2′′)]; 23.9 [C(3′), C(3′′)]; 20.4 [C(4′), C(5′), C(4′′), C(5′′)]; MS, *m/z* (%): 278 (57) [M^+^], 235 (87), 222 (100), 221 (51), 208 (15), 194 (14), 179 (27), 163 (23), 152 (15), 138 (14), 125 (18), 82 (17), 68 (27), 55 (24); HRMS (ESI TOF-MS): Calcd. for C_16_H_27_N_2_O_2_ 279.2067; found: 279.2069.

The other compounds **12-16**, **18-23 **were prepared employing a procedure similar to that described for **17**, and yields are presented in Scheme 1. All the compounds were fully characterized by IR, ^1^H- and ^13^C-NMR and mass spectrometry. The structural characterization of compounds **12**, **13**, **14**, **18**, **22**, and **23 **has already been described [34,45,46]. Compounds **15**, **16**, **17**, **20**, and **21** have already been synthesized, although their complete spectroscopic data were not given [47,48,49,50,51]. Structures for **15**, **16**, **17**, **20**, and **21** and the remaining compounds are supported by the following spectroscopic data:

*2,5-Bis(isobutylamino)-1,4-benzoquinone* (**15**). Red crystals. Purified by column chromatography, eluent hexane/diethylether 1:1 (*v/v*). Mp 188.4–191.5 ºC; IR: 3,274 (NH), 2,953, 2,869, 1,645, 1,554, 1,491, 1,443, 1,366, 1,255, 1,065, 815, and 723 cm^-1^; ^1^H-NMR δ: 6.71 (br. s, NH); 5.37 [s, H-C(3), H-C(6)]; 2.97 [t, *J* = 6.6 Hz, 2 H-C(1′), 2 H-C(1′′)]; 1.90-2.04 [m, H-C(2′), H-C(2′′)]; 0.98 [d, *J* = 6.6 Hz, 3 H-C(3′), 3 H-C(4′), 3 H-C(3′′), 3 H-C(4′′)]; ^13^C-NMR δ: 178.3 [C(1), C(4)]; 151.8 [C(2), C(5)] 93.0 [C(3), C(6)]; 50.3 [C(1′), C(1′′)]; 28.0 [C(2′), C(2′′)]; 20.5 [C(3′), C(4′), C(3′′), C(4′′)]; MS, m/z (%): 250 (84) [M^+^], 235 (13), 207 (100), 193 (40), 165 (28), 164 (31), 151 (60), 138 (23), 123 (20), 67 (21), 53 (46); HRMS (ESI TOF-MS): Calcd. for C_14_H_23_N_2_O_2_ 251.1754; found: 251.1756.

*2,5-Bis-(pentylamino)-1,4-benzoquinone *(**16**). Red crystals. Purified by column chromatography, eluent hexane/dichloromethane 1:2 (*v/v*). Mp 134.6–137.1 ºC; IR: 3,263 (NH), 2,957, 2,927, 2,857, 1,644, 1,553, 1,497, 1,463, 1,369, 1271, and 812 cm^-1^; ^1^H-NMR (* indicates assignments that could be reversed) δ: 6.62 (br. s, NH), 5.28 [s, H-C(3), H-C(6)]; 3.12 [q, *J* = 6.8 Hz, 2 H-C(1′); 2 H-C(1′′)]; 1.59-1.68 [m, 2 H-C(2′), 2 H-C(2′′)]; 1.21-1.38 [m, 2 H-C(3′), 2 H-C(4′), 2 H-C(3′′), 2 H-C(4′′)]; 0.86-0.91 [m, 3 H-C(5′), 3 H-C(5′′)]; ^13^C-NMR δ: 177.1 [C(1), C(4)]; 150.4 [C(2), C(5)]; 91.6 [C(3), C(6)]; 41.6 [C(1′), C(1′′)]; 28.1 [C(3′), C(3′′)]^*^; 27.0 [C(2′), C(2′′)]; 21.3 [C(4′), C(4′′)]^*^; 13.0 [C(5′), C(5′′)]; MS, *m/z* (%): 278 (100) [M^+^], 235 (73), 223 (30), 207 (40), 193 (46), 179 (28), 165 (62), 164 (19), 151 (45), 138 (23), 137 (23), 110 (23), 67 (54), 54 (56); HRMS (ESI TOF-MS): Calcd. for C_16_H_27_N_2_O_2_ 279.2067; found: 279.2070.

*2,5-Bis-(decylamino)-1,4-benzoquinone *(**19**): Red crystals. Purified by column chromatography, eluent hexane/diethylether 2:1 (*v/v*). Mp 126.4–127.3 ºC; IR: 3,256 (NH), 2,954, 2,917, 2,848, 1,644, 1,554, 1,503, 1,456, 1,365, 1,291, and 683 cm^-1^; ^1^H-NMR δ: 6.62 (br. s, NH); 5.30 [s, H-C(3), H-C(6)]; 3.13 [q, *J* = 6.6 Hz, 2 H-C(1′), 2 H-C(1′′)]; 1.50-1.70 [m, 2 H-C(2′), 2 H-C(2′′)]; 1.15-1.40 [m, 14 H-C(3′-9′), 14 H-C(3′′-9′′)]; 0.88 [t, *J* = 6.6 Hz, 3 H-C(10′), 3 H-C(10′′)]; ^13^C-NMR (^*^ indicates assignments that could be reversed) δ: 178.3 [C(1), C(4)]; 151.6 (C(2), C(5)]; 92.8 [C(3), C(6)]; 42.8 (C(1′), C(1′′)]; 32.1 [C(2′), C(2′′)]^*^; 29.7 [C(8′), C(8′′)]^*^; 29.7 [C(3′), C(3′′)]^*^; 29.5 [C(4′), C(4′′)]^*^; 29.4 [C(5′), C(5′′)]^*^; 28.5 [C(6′), C(6′′)]^*^; 27.2 [C(7′), C(7′′)]^*^; 22.9 [C(9′), C(9′′)]^*^; 14.4 [C(10′), C(10′′)]; MS, *m/z* (%): 418 (100) [M^+^], 362 (35), 333 (11), 305 (31), 165 (25), 162 (27), 151 (17), 138 (17), 68 (20), 54 (30). HRMS (ESI TOF-MS): Calcd. for C_26_H_47_N_2_O_2_ 419.3632; found: 419.3637.

*2,5-Bis-(1-(2-aminoethyl)piperidino)-1,4-benzoquinone *(**20**). Red crystals. Purified by column chromatography, eluent hexane/methanol 1:3 (*v/v*). Mp 164.2–165.5 ºC; IR: 3,291 (NH), 3,251 (NH), 2,935, 2,849, 1,641, 1,551, 1,493, 1,462, 1,364, 1,289, 1,223, 1,126, 993, and 693 cm^-1^; ^1^H-NMR δ: 7.04 (br. s, NH); 5.30 [s, H-C(3), H-C(6)]; 3.19 [q, *J* = 6.0 Hz, 2 H-C(1′), 2 H-C(1′′)]; 2.58 [t, *J* = 6.0 Hz, 2 H-C(2′), 2 H-C(2′′)]; 2.34-2.42 [m, 2 H-C(3′), 2 H-C(7′), 2 H-C(3′′), 2 H-C(7′′)]; 1.59 [quint., *J* = 5.5 Hz, 2 H-C(4′), 2 H-C(6′), 2 H-C(4′′), 2 H-C(6′′)]; 1.40-1.44 [m, 2 H-C(5′), 2 H-C(5′′)]; ^13^C-NMR δ: 178.5 [C(1), C(4)]; 151.6 [C(2), C(5)]; 93.3 [C(3), C(6)]; 56.11 [C(2′), C(2′′)]; 54.5 [C(3′), C(7′), C(3′′), C(7′′)]; 39.3 (C(1′), C(1′′)]; 26.1 [C(4′), C(6′), C(4′′), C(6′′)]; 24.5 [C(5′), C(5′′)]; MS, m/z (%): 360 (2) [M^+^], 99 (11), 98 (100), 70 (6), 55 (14). HRMS (ESI TOF-MS): Calcd. for C_20_H_33_N_4_O_2_ 361.2598; found: 361.2599.

*2,5-Bis-(4-(2-aminoethyl)morfoline)-1,4-benzoquinone *(**21**). Red crystals. Purified by column chromatography, eluent hexane/ethanol 1:4 (v/v). Mp 186.8–188.0 ºC; IR: 3,354 (NH), 2,869, 2,811, 1,645, 1,613, 1,495, 1,455, 1,354, 1,293, 1,115, 1,026, and 809 cm^-1^; ^1^H-NMR δ: 7.00 (br. s, NH); 5.28 [s, H-C(3), H-C(6)]; 3.72 [t, *J* = 4.5 Hz, 2 H-C(4′), 2 H-C(5′), 2 H-C(4′′), 2 H-C(5′′)]; 3.20 [q,*J* = 6.0 Hz, 2 H-C(1′), 2 H-C(1′′)]; 2.64 [t, *J* = 6.0 Hz, 2 H-C(2′), 2 H-C(2′′)]; 2.47 [t, *J* = 4.5 Hz, 2 H-C(3′), 2 H-C(6′), 2 H-C(3′′), 2 H-(6′′)]; ^13^C-NMR (CDCl_3_) δ: 178.3 [C(1), C(4)]; 151.0 [C(2), C(5)]; 93.1 [C(3), C(6)]; 66.8 [C(4′), C(5′), C(4′′), C(5′′)]; 55.5 [C(2′), C(2′′)]; 53.2 [C(3′), C(6′), C(3′′), C(6′′)]; 38.5 [C(1′), C(1′′)]; MS, m/z (%): 364 (3) [M^+^], 101 (10), 100 (100), 70 (8), 56 (24); HRMS (ESI TOF-MS): Calcd. for C_18_H_29_N_4_O_4_ 365.2183; found: 365.2186.

### 3.3. Biological assays

#### 3.3.1. Phytotoxic activity

The phytotoxic activity of the 2,5-bis(alkylamino)-1,4-benzoquinones **12-23** was preliminarily evaluated as the ability of these compounds to interfere with the radicle growth of the cultivars *Sorghum bicolor* and *Cucumis sativus*. For these biological assays, stock solutions at 1.0 × 10^-3^ mol/L were prepared as follows: each compound **12-23** was dissolved in xylene (84 μL), Tween 80 surfactant (127 μL) and pentan-3-one (42 μL). The resultant suspension was shaken for 1 min and then transferred to a volumetric flask and the volume supplemented with water to 88 mL. The resultant suspension was sonicated for 5 min.

#### 3.3.2. Assay of radicle elongation on Petri dishes

This biological assay was carried out as previously described [52,53]. Groups of seven pregerminated plants of *S. bicolor *(purchased from Geneze Company, Paracatu, Minas Gerais State, Brazil) were placed in Petri dishes (i.d. = 9 cm) containing washed sand (660 g) and the solution (88 mL) containing the compound to be evaluated. The Petri dishes were sealed with Parafilm, incubated at 28 °C, and inclinated at 75°. After 24 h and 48 h, the radicle length was measured to the nearest millimeter. All treatments were replicated four times in a completely randomized design. The percentage of radicle growth inhibition was calculated in relation to the root length of the control. Positive values represent inhibition and negative values correspond to stimulation. The data were analyzed using Tukey′s test at 0.05 probability level [54]. This biological assay was also conducted with *C. sativus* L. (purchased from ISLA Company, Porto Alegre, Rio Grande do Sul State, Brazil), *B. decumbens* (Marangatú Company, Ribeirão Preto, São Paulo State) and *I. grandifolia* (Agro Cosmos, Engenheiro Coelho, São Paulo State).

#### 3.3.3. Measurement of the ATP synthesis

Intact chloroplasts were isolated from spinach leaves (*Spinacea oleracea *L.) obtained from local market as previously described [55]. Chloroplasts were suspended in the following medium: 400 mmol/L sucrose, 5 mmol/L MgCl_2_, 10 mmol/L KCl, and buffered with 0.03 mol/L Na^+^-tricine at pH 8.0. They were stored as a concentrated suspension in the dark for 1 h at 0 °C. Intact chloroplasts were efficiently lysed to yield free thylakoids prior to each experiment by incubating them in the following electron transport medium: 100 mmol/l sorbitol, 10 mmol/L KCl, 5 mmol/L KCN, and 30 mmol/L tricine buffer (pH 8 with the addition of KOH). Chlorophyll concentration was measured spectrophotometrically as reported [56].

ATP synthesis was measured as the pH rise from 8.000 to 8.100 using a microelectrode connected to a Corning potentiometer with expanded scale. The pH change was recorded with a Gilson recorder. The reaction medium contained 100 mmol/L sorbitol, 5 mmol/L MgCl_2_, 10 mmol/L KCl and 1 μmol/L K^+^-tricine at pH 8.0 in the presence of 1 mmol/L ADP and 3 mmol/L KH_2_PO_4_. Methylviologen (0.05 mol/L) was added as electron acceptor for the Hill reaction. The effect of compounds **12**, **13** and **23 **on the ATP synthesis was evaluated at 100 μmol/L, 200 μmol/L, 300 μmol/L, 400 μmol/L, and 500 μmol/L. All mixtures were illuminated with actinic light of a projector lamp (GAP 2660) passed through a 5 cm aqueous solution of 2% CuSO_4_ as filter [57].

### 3.4. Cytotoxicity screening

#### 3.4.1. Cell lines and cell cultures

The cytotoxicity of compounds **12-14** and **16-23 **was tested against HL-60 (human leukemia), MDA-MB-435 (human breast cancer), HCT-8 (human colon) and SF-295 (human central nervous system) cell lines obtained from the National Cancer Institute (Bethesda, MD, USA). Cells cultured in RPMI 1640 medium supplemented with 10% fetal bovine serum, 2 mmol/L glutamine, 100 U/mL penicillin, 100 μg/mL streptomycin at 37 °C under a 5% CO_2_ atmosphere.

Heparinized blood (from healthy, non-smoker donors who had not taken any drug at least 15 days prior to sampling) was collected and Peripheral Blood Mononuclear Cells (PBMC) were isolated by a standard method of density-gradient centrifugation over Histopaque-1077. PBMC were washed and resuspended in RPMI 1640 medium supplemented with 20% fetal bovine serum, 2 mmol/L glutamine, 100 U/mL penicillin, 100 μg/mL streptomycin at 37 °C under a 5% CO_2_ atmosphere. Phytohemaglutinin (2 %) was added at the beginning of culture. After 24 h of culture, cells were treated with the test compounds **12-14** and **16-23**.

#### 3.4.2. MTT assay

Tumor cell growth was quantified by the ability of living cells to reduce the yellow dye 3-(4,5-dimethyl-2-thiozolyl)-2,5-diphenyl-2H-tetrazolium bromide (MTT) to a purple formazan product [38]. For the experiments, cells were plated in 96-well plates (0.7 × 10^5^ cells/mL for MDA-MB-435, HCT-8 and SF-295 cell lines, and 0.3 × 10^6^ cells/mL for leukemia cells). After 24 h, the test compounds (0.39 to 25 μg/mL) dissolved in DMSO (0.1%), were added to each well and incubated for 72 h. DMSO (0.1%) and doxorubicin were used as negative and positive controls, respectively. Thereafter, the plates were centrifuged and then the medium was replaced by fresh medium (150 μL) containing 0.5 mg/mL MTT. Three hours later, the MTT formazan product was dissolved in 150 µL DMSO, and absorbance was measured using a multiplate reader (Spectra Count, Packard, Ontario, Canada). Drug effect was quantified as the percentage of control absorbance of the reduced dye at 595 nm in relation to control wells.

#### 3.4.3. Alamar Blue assay

In order to investigate selectivity of compounds toward a normal proliferating cell, the Alamar Blue assay was performed with PBMC after 72 h drug exposure [39]. Briefly, PBMC were plated in 96-well plates (3 × 10^5^ cells/well in 100 µL of medium). After 24 h, the compounds (0.09–25 µg/mL) dissolved in DMSO were added to each well (using the HTS - high-throughput screening - Biomek 3000 - Beckman Coulter, Inc., Fullerton, California, USA) and incubated for 72 h. Doxorubicin was used as positive control. Twenty four hours before the end of the incubation, 10 µL of stock solution (0.312 mg/mL) of the Alamar Blue (resazurin - Sigma Aldrich Co. - St. Louis, MO, USA) was added to each well. The absorbance was measured using a multiplate reader (DTX 880 Multimode Detector, Beckman Coulter, Inc). The drug effect was quantified as the percentage of control absorbance at 570 nm and 595 nm.

## 4. Conclusions

A series of 2,5-bis(alkylamino)-1,4-benzoquinone analogues of sorgoleone (**1**) were synthesized and biologically evaluated in terms of their phytotoxicity and cytotoxicity. The assessment of phytotoxicity revealed that the compounds can act as plant growth regulators with various patterns of activity. Moreover, differences were noticed concerning the effects of the compounds on monocotyledonous and dicotyledonous species. Further investigation on the phytotoxicity of the 2,5-bis(alkylamino)-1,4-benzoquinones showed that they are capable of inhibiting the ATP synthesis in isolated chloroplasts. The cytotoxicity assays revealed that the compounds 2,5-bis(ethylamino)-1,4-benzoquinone **12** and 2,5-bis(1-(2-aminoethyl)piperidino)-1,4-benzoquinone **20** exhibited activity against all of the human cell lines used in the biological evaluation. The 2,5-bis(ethylamino) derivative **12** exhibited the most selective cytotoxicity against the tumor cell lines tested and is not toxic to normal cells.
